# Densely Packed Microgoblet Laser Pairs for Cross‐Referenced Biomolecular Detection

**DOI:** 10.1002/advs.201500066

**Published:** 2015-06-08

**Authors:** Uwe Bog, Falko Brinkmann, Sentayehu Fetene Wondimu, Tobias Wienhold, Sarah Kraemmer, Christian Koos, Heinz Kalt, Michael Hirtz, Harald Fuchs, Sebastian Koeber, Timo Mappes

**Affiliations:** ^1^ Institute of Nanotechnology Karlsruhe Nano Micro Facility (KNMF)Karlsruhe Institute of Technology (KIT)76128KarlsruheGermany; ^2^ Institute of Microstructure Technology (IMT)Karlsruhe Institute of Technology (KIT)76128KarlsruheGermany; ^3^ Physical Institute and Center for Nanotechnology (CeNTech)Westfälische Wilhelms‐Universität48149MünsterGermany; ^4^ Institute of Applied Physics (APH)Karlsruhe Institute of Technology (KIT)76128KarlsruheGermany; ^5^ Institute of Photonics and Quantum Electronics (IPQ)Karlsruhe Institute of Technology (KIT)76128KarlsruheGermany; ^6^ Carl Zeiss AG, Corporate Research and TechnologyCarl‐Zeiss‐Promenade 1007745JenaGermany

**Keywords:** aligned microcontact stamping, biosensing, microlasers, multiplexed surface functionalization, whispering gallery mode resonators

## Abstract

**Microgoblet laser pairs are presented for cross‐referenced on‐chip biomolecular sensing.** Parallel readout of the micro­lasers facilitates effective mutual filtering of highly localized refractive index and temperature fluctuations in the analyte. Cross‐referenced detection of two different types of proteins and complete chemical transducer reconfiguration is demonstrated. Selective surface functionalization of the individual lasers with high spatial accuracy is achieved by aligned microcontact stamping.

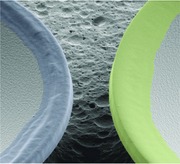

In the recent past research interest in photonic whispering gallery mode (WGM) structures for label‐free molecular detection has strongly increased.^1^, ^2^ Manifold geometries have been demonstrated as compact and highly sensitive devices, e.g., microtoroids,^3^, ^4^ microspheres,^5^, ^6^ microrings,^7^, ^8^ capillaries,^9^, ^10^ and optical fibers.^11^, ^12^ Most WGM structures provide outstandingly narrow spectral resonance features making them very promising for high‐resolution detection of molecular binding events to their surfaces. To address single sensors, most prominently evanescent field coupling with a tapered optical fiber is used,^1^, ^2^, ^3^, ^4^, ^5^, ^9^, ^13^ offering good coupling efficiency and signal quality. However, this configuration requires high alignment effort and mechanical stability on the nanometer scale throughout the whole sensing experiment. Therefore, simultaneous probing and readout of more than one sensor remains very challenging. In particular, chip‐based resonator geometries suffer from this drawback as these structures are usually located in very close proximity to the substrate surface.^1^, ^13^, ^14^ Besides ring resonators with integrated waveguides,^7^, ^8^ no other chip‐based WGM sensing platform capable of simultaneous signal processing of multiple microcavities has been demonstrated so far. However, this capability is crucial for real‐world applications. It enables the compensation of sensing signal deteriorations originating, e.g., from localized density and temperature fluctuations or from nonspecific molecular adsorption events. A sole temperature referencing can be performed with one single WGM transducer, but requires rather complex transducer preparation methods and well controlled high resolution WGM readout schemes.^1^, ^15^ Still, signal disturbances from refractive index fluctuations or nonspecific molecular binding can solely be filtered out by the use of at least one additional reference transducer.

A promising solution to these restrictions are WGM microgoblet lasers fabricated out of polymers.^16^, ^17^, ^18^, ^19^, ^20^ Here, the implementation of lasing dyes into the optical cavity results in active sensing elements, which can be probed and read out with very low alignment requirements via free‐space optics. Since fabrication is performed photolithographically, the sensors can be realized cost‐efficiently and with very high device density and throughput on the wafer scale.^16^ High lateral patterning accuracy allows a highly defined and reproducible geometrical design of the WGM cavities. Hence, defined cavity configurations can easily be realized, with each individual resonator exhibiting a distinct set of WGMs. Since the cavities can be structured very close to each other a more efficient signal referencing is possible as even highly localized disturbances may be accounted for.

In this work we present a sensing platform for facile cross‐referenced detection of multiple different molecules. We utilize high‐*Q* microgoblet lasers fabricated from dye‐doped poly(methyl methacrylate) (PMMA)^16^ structured pairwise in close proximity to each other, allowing simultaneous free space optical pumping and readout. The individual laser cavities exhibit different radii and hence support mode sets with different free spectral ranges (FSR). Assignment of specific modes from the complex spectrum to each resonator is therefore straightforward. Utilizing the parallel readout of both resonators we discuss the cross‐referencing capability in detail. By using one cavity as a reference at an assigned time period during the sensing experiment, signal disturbances arising from temperature and refractive index fluctuations can mutually be compensated. Additionally, we show the detection of two different proteins with the very same microlaser pair. The microlasers are selectively surface functionalized with very high lateral accuracy with two different headgroup‐modified phospholipid inks by aligned microcontact stamping (AμCS).^18^ One of the utilized inks comprises chemical end groups for reversible microfluidic coupling and decoupling of linker‐tagged proteins, enabling an in situ reconfiguration of the respective microlaser during the sensing experiment. By the subsequent detection of polyhistidine‐tagged green fluorescent protein (his‐GFP) with the very same microlaser we show how referencing of the sensing signals results in a fivefold improved signal reproducibility.

To ensure efficient simultaneous pumping and readout, the individual microgoblet lasers of the microlaser pairs had a lateral distance of around 3 μm. Due to their different cavity radii, the microgoblets support sets of lasing modes with different FSRs. Explicit assignment of the lasing peaks to the corresponding microlaser enables correlation of spectral shifts to molecular binding events occurring at the respective resonator circumference. The surface functionalization of the microlaser pairs is performed by AμCS, as depicted in **Figure** **1**a–d. As ink stamp pad a microscope glass slide is utilized, onto which phospholipid inks with different functional headgroups are deposited as laterally confined films. Individual microlasers are selectively inked utilizing the interface areas between the laterally confined ink films and the bare glass. Therefore the microlaser to be inked is aligned on the ink film side and the second laser on the area of the bare glass of an ink spot interface. For all presented experiments the individual cavities of each microlaser pair are coated with different phospholipid inks to functionalize them with different molecular acceptors. The phospholipid transfer is typically limited to the circumference of the lasers as solely the goblet rims are brought into contact with the lipid ink spots (Figure 1e,h).

**Figure 1:**
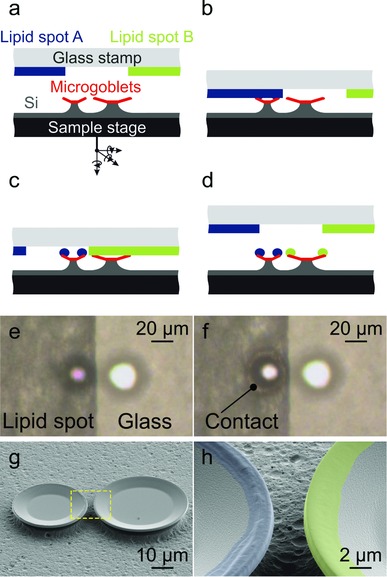
a–d) Schematic principle of the AμCS inking process. The glass stamp pad comprises two different phospholipid ink films and is glued upside down onto a custom made holder. A piezo‐actuated six‐axis translational stage is utilized for mechanical alignment of the microlaser chips relative to the stamp pad. The single microlasers are selectively coated by aligning the symmetry axis of the laser pair perpendicular to the respective lipid film interface, with the respective targeted laser located on the inked and the second laser on the noninked side of the interface. The ink transfer procedure is visually controlled through the stamp pad via a CCD camera. e) In situ picture of the inking process prior stamp pad contact. f) Inking of the left goblet. A color change along the circumference indicates physical contact between ink film and laser. g) SEM image of an inked microlaser pair. h) Close‐up picture of the boxed area in (g) with lipid ink functionalization demarked by false color. The phospholipids are clearly limited to the circumferences of the lasers.

Optical characterization is performed by using a laboratory spectrograph with a Peltier‐cooled camera, equipped with a 2D charge‐coupled device (CCD) pixel array (**Figure** **2**a). Detailed simultaneous analysis of the lasing modes is realized by aligning the symmetry axis of the goblet pairs along the entrance slit of the spectrograph (Figure 2a, inset picture). Figure 2b shows the combined lasing spectrum of a functionalized microlaser pair immersed in phosphate buffered saline (PBS), recorded by full vertical binning (FVB, columnwise integration of the CCD pixel intensity signals). The assignment of a specific lasing mode to the respective microcavity can be performed by considering the FSRs Δ*λ*_FSR_ of the resonators. With the smaller FSR set of modes (Δ*λ*_FSR‐L_ = 1.71 nm) belonging to the larger radius microcavity and vice versa (Δ*λ*_FSR‐S_ = 2.17 nm), individual lasing peaks of each microlaser can be identified. For verification of this approach, Figure 2b shows the spectrum of each microgoblet recorded individually.

**Figure 2:**
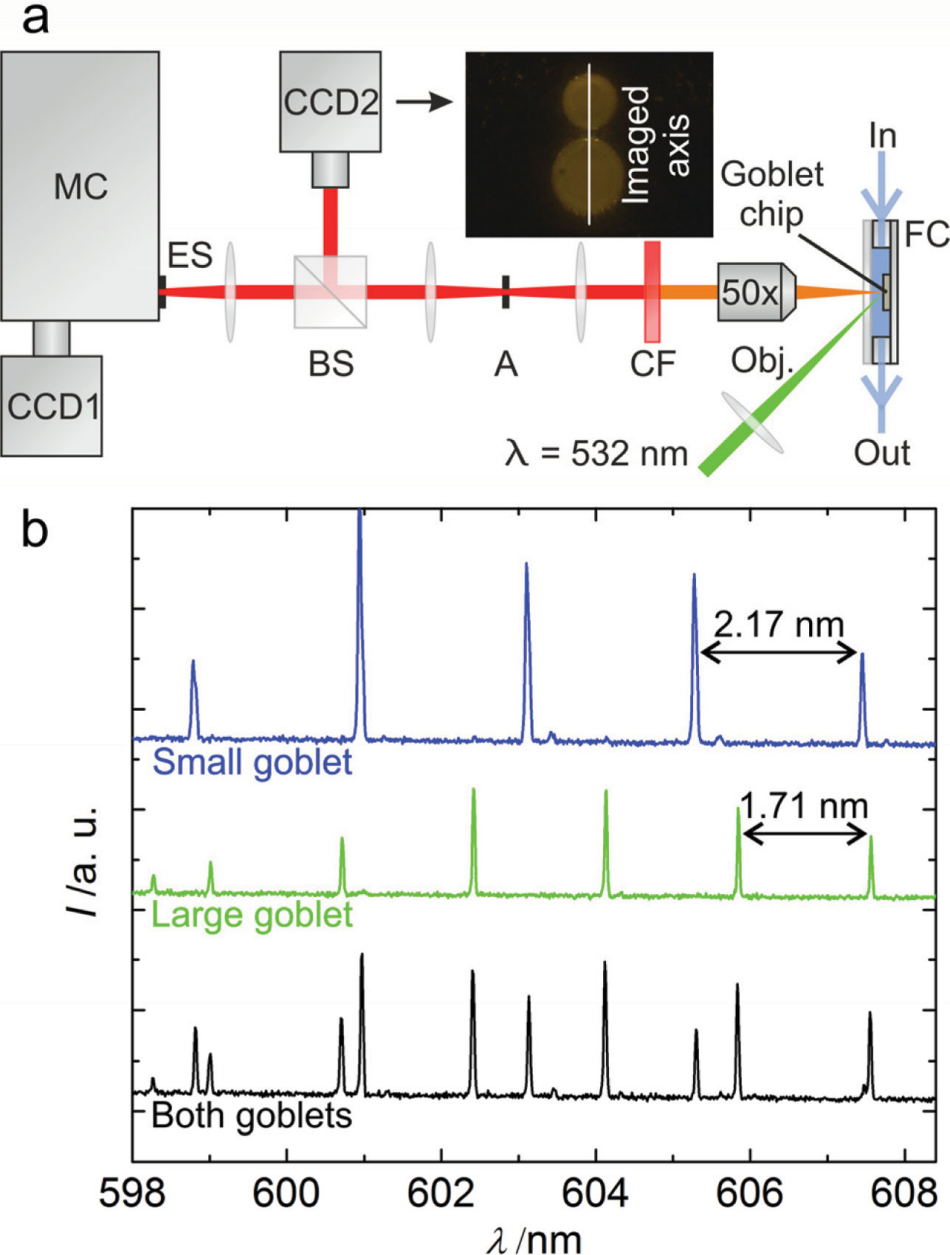
a) Optical characterization setup: One microlaser pair, located in a microfluidic chamber (FC), is aligned with its symmetry axis along the entrance slit (ES) of a spectrograph (monochromator (MC), equipped with a 2400 L mm^−1^ grating and a Peltier‐cooled CCD camera with 1024 horizontal and 256 vertical pixels (CCD1)), to investigate the emission spectrum along this axis (see inset picture). Optical pumping is performed with 150–200 nJ per pulse under an incident angle of 45° by a frequency‐doubled Nd:YVO4 laser (*λ* = 532 nm, 10 ns pulse duration, 20 Hz repetition rate, 100 μm spot size). The emitted light is collected with a 50× microscope objective (MO). The pump laser wavelength is filtered out by a long pass filter (CF). For parallel visualization of the laser pair a beamsplitter (BS) and a CCD camera (CCD2) are utilized. Optionally the investigated region can be laterally limited by an aperture A, to select an individual goblet. b) FVB spectrum of a microgoblet pair immersed in PBS as well as the spectrum of each individual laser when pumped individually. The microlasers are distinguished by the resonator dependant free spectral ranges of their lasing mode sets.

Within any biomolecular detection experiment, local temperature and refractive index fluctuations of the analyte typically result in significant sensor signal deterioration, in particular when the measurements are carried out outside of a controlled laboratory environment. For example, during point‐of‐care diagnostics ambient temperature can hardly be controlled. Here, incident sunlight or the body temperature of the operator or the patient may induce considerable signal deviations. Additionally, refractive index fluctuations induced by the use of different analyte solutions or nonspecific molecule adsorption can further impair the sensing result.

To demonstrate the referencing capability of the microlaser pairs, we performed a molecular detection experiment mimicking these typical signal disturbances (**Figure** **3**). For this purpose, the small diameter microlaser was functionalized with a phospholipid ink consisting of 4 mol% Biotinyl Cap PE in 1,2‐dioleoyl‐*sn*‐glycero‐3‐phosphocholine (DOPC) (later referred to as biotin ink) to obtain a sensor for streptavidin. To configure the large diameter laser as a passivated reference sensing element it was coated with pure DOPC and did therefore not provide any molecular binding sites. The goblet chip was then mounted into the microfluidic chamber of the optical characterization setup. To induce temperature changes a Peltier element was glued to the metal frame of the microfluidic chamber. Temperature monitoring was established by placing a type K thermo couple temperature sensor into close vicinity to the chip. Prior to the sensing experiment the functionalized goblet pair was kept in a 0.5% bovine serum albumin (BSA) solution for 30 min to further suppress unspecific binding. At the beginning of the streptavidin detection the lasers were immersed in pure water at room temperature. The streptavidin (12 μg mL^−1^) injected after 1 min was diluted in PBS, so that the lasing modes of both lasers experienced an abrupt redshift due to the slightly higher refractive index of the PBS (Δ*n* = +0.0017). During the streptavidin incubation the temperature of the analyte was increased first by +0.1 K at time point 12 min and then by additional +1 K at 14.5 min, to simulate arbitrary temperature drifts. At 16.5 min the Peltier element was switched off, to let the analyte cool down back to room temperature. Finally, the fluidic chamber was purged with water. Direct analysis of the temporal spectral lasing mode shifts of the biotinylated laser (Figure 3, blue curve) shows considerable signal disturbances induced by the temperature changes. While the signal of the reference goblet (Figure 3, green curve) shows analogous disturbances, it additionally reveals the refractive index change induced by the PBS, which is not intuitively visible by the sole investigation of the biotinylated laser's sensing signal.

**Figure 3:**
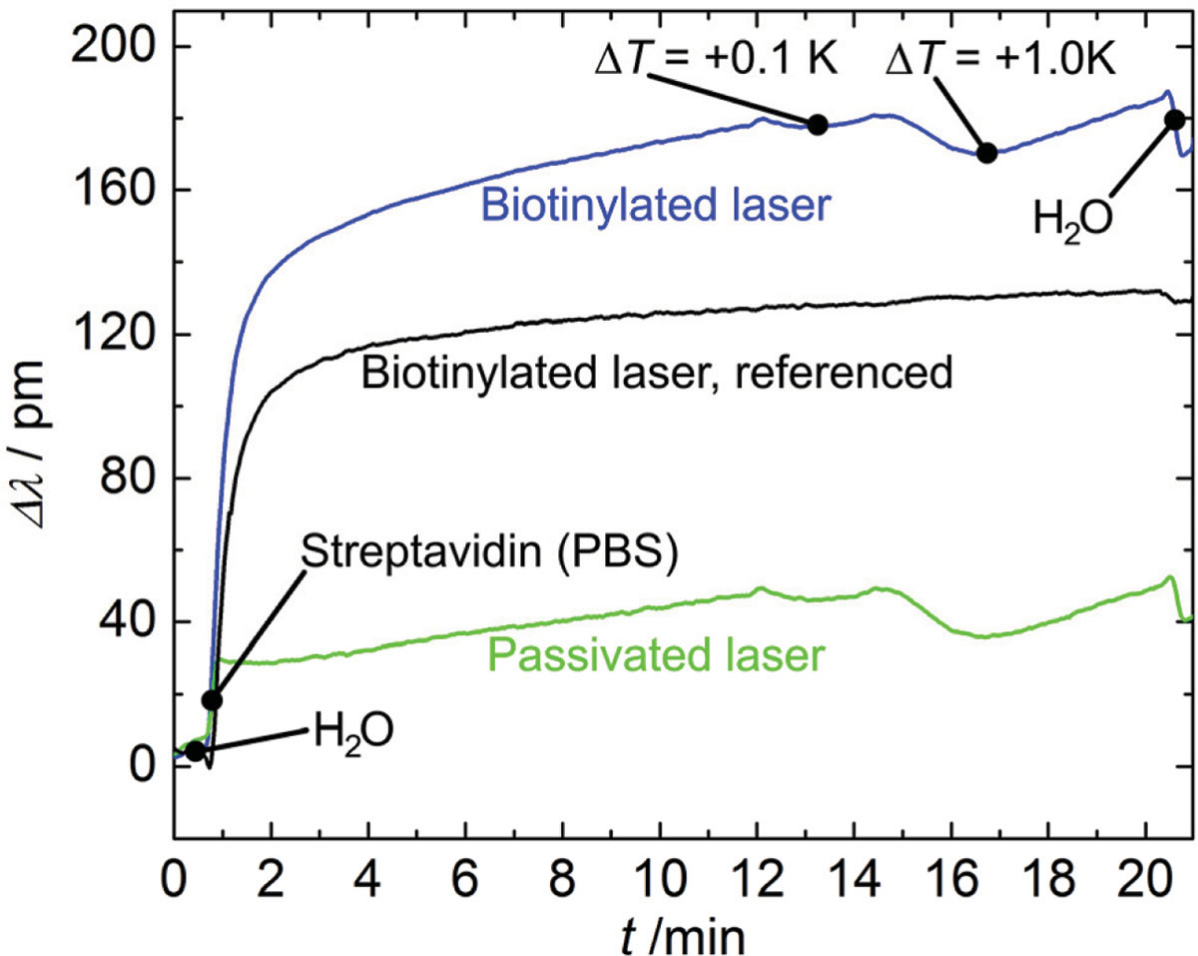
Referencing capability demonstration by a streptavidin binding experiment. During the experiment the refractive index and temperature of the analyte are perturbed at defined time points. The figure shows the acquainted signals of the biotinylated and DOPC‐passivated goblet as well as the referenced detection signal of the biotinylated laser.

To filter out all these signal disturbances, the signal of the reference goblet can be utilized as a straightforward “reference baseline.” For this purpose, the temperature and refractive index sensitivities (temperature sensitivity (TS) and bulk refractive index sensitivity (BRIS)) of the individual microgoblets must be known. The filtering can then be performed by an accordingly weighted point‐to‐point subtraction of the reference goblet signal from the sensing goblet signal. In prior experiments, the BRIS of the individual microlasers was experimentally assessed by exposing unfunctionalized microlaser pairs to different concentrations of glycerol in water and evaluating the resulting spectral shifts of the lasing modes. The average BRIS of the small diameter laser modes in nm per refractive index unit (RIU) was 20.07 nm RIU^−1^ (standard deviation 0.52 nm RIU^−1^). For the large diameter laser the measured BRIS was 14.80 nm RIU^−1^ (standard deviation 0.20 nm RIU^−1^). To characterize the TS the temperature was increased in discrete steps while the goblet pairs were kept in water. The average TS of the small diameter laser modes was −14.18 pm K^−1^ (standard deviation 0.25 pm K^−1^). The large diameter laser TS was measured to −14.99 pm K^−1^ (standard deviation 0.27 pm K^−1^).

Since the BRIS and TS of the two microlasers are different, the raw signal of the reference laser Δ*λ*_ref,raw_ has to be adequately weighted before it can be utilized for referencing. The weighted reference signal is then subtracted from the raw signal Δ*λ*_sens,raw_ of the sensing laser to obtain the referenced signal Δ*λ*_sens,corr,t_

(1) $$\begin{array}{l} \Delta \lambda_{\mathrm{sens},\mathrm{corr},t}=\sum_{t=\Delta t}^{n\Delta t}\left(\frac{d(\Delta \lambda_{\mathrm{sens},\mathrm{raw},t})}{dt}-\frac{S_{\mathrm{sens},t}}{S_{\mathrm{ref},t}}\frac{d(\Delta \lambda_{\mathrm{ref},\mathrm{raw},t})}{dt}\right)\Delta t\\ \;\;\;\;\;+\Delta \lambda_{\mathrm{sens},\mathrm{corr},t=0} \end{array}$$

(2) $$\Delta \lambda_{\mathrm{sens},\mathrm{corr},t=0}=\Delta \lambda_{0}$$

*S*_sens_ and *S*_ref_ are either the TS or BRIS of the sensing, and the referencing laser to be considered at a time, respectively; Δ*t* is the acquisition time; Δ*λ*_0_ represents the first acquisition data point for offset correction.

The referencing leads to significant improvement of the biotinylated laser's sensing signal (Figure 3, black curve). Besides the accurate filtering of the temperature induced signal contributions, also the obscure signal offset triggered by the refractive index of the PBS is compensated. The referenced sensor signal of the biotinylated laser now delivers the generally expected exponentially saturating streptavidin binding curve.^21^

If both microlasers provide different types of binding sites, mutual signal filtering can be performed (cross‐referencing). The cross‐referenced detection of two different kinds of proteins is demonstrated in **Figure** **4**. Additionally, within this experiment a targeted reconfiguration of one of the sensor elements is performed. For this purpose, the small diameter laser was functionalized with 25 mol% 1,2‐dioleoyl‐sn‐glycero‐3‐{[*N*(5‐amino‐1‐carboxypentyl)iminodiacetic acid]succinyl} nickel salt (DOGS‐NTA‐Ni) in DOPC to decorate the microgoblet with nickel (Ni) chelating headgroup components for selective binding of his‐tagged molecules. As Ni/his‐tag binding is reversible this ink allows facile multiple binding and removal of his‐tagged acceptor molecules, enabling an in situ sensor reconfiguration with different types of biorecognition elements. The large diameter microlaser was coated with the biotin ink. The molecular binding experiment reveals the high lateral accuracy of the surface functionalization by AμCS. Despite the short lateral distance of the single microlasers (≈3 μm), their individual lasing modes exclusively respond to the specific binding of the respective target molecules (streptavidin and his‐GFP (green fluorescent protein)) (Figure 4a). Figure 4b shows that the binding curves of both microlasers are superposed by nonspecific signal contributions, which can be attributed to temperature and refractive index changes induced by the fluid injections. For example, the his‐GFP bound to the Ni‐chelated microlaser in a first incubation round (from 1.5 to 6.5 min) is removed by a solution of imidazole in PBS (7.5 to 7.75 min) to re‐avail the Ni‐chelate end groups for a subsequent second binding round (9.25 to 14.25 min) (Figure 4b, blue curve). During this reconfiguration process a considerable disturbance of the Ni‐chelated microlaser signal can be observed. First, the superposed step function during the blueshift of the Ni‐chelated microlaser signal is the result of the higher refractive index of the imidazole solution (Δ*n* = +0.0016, compared to the PBS buffer used for the his‐GFP), as indicated by the peak in the biotinylated laser signal (Figure 4b, green curve). Second, after the reconfiguration the biotinylated laser signal is offset by ≈4.8 pm, indicating a slight nonspecific adsorption of imidazole molecules to both microlasers. Furthermore, the temporally bluedrifting signal of the biotinylated laser during both GFP incubation rounds indicates slight temperature increase induced at each injection event. Due to the sign of the drifts refractive index disturbances based on nonspecific his‐GFP adsorption can be excluded. This indicates the generally very good protein repulsion characteristics of the phospholipid inks already observed in previous publications.^18^, ^22^ As a result of all these disturbances, the sensing signal generated by the Ni‐chelated microlaser during the first and the second his‐GFP binding round shows significant differences. Besides the signal offset, the two binding curves saturate at significantly different values (first his‐GFP incubation round: 71.86 pm; second incubation round: 83.61 pm). For each analyte incubation the nonbinding specific signals occurring at the laser with the respective incompatible molecular acceptors can be utilized once more as a reference for the signals of the matching laser. The mutual referencing of the sensing signals leads to the filtered molecular binding signals for both microlasers (Figure 4b, black curves). A general improvement of the detection result is intuitively visible by the now constant temporal sensing signals prior and after the respective streptavidin/his‐GFP injections, indicating accurate correction of the temperature induced drifts. Furthermore, the refractive index disturbance as well as the signal offset triggered by the nonspecific imidazole adsorption has fully been compensated. Eventually, the binding curves of the Ni‐chelated goblet of both his‐GFP incubation rounds now show a reduced deviation from previously 16.4% to now 3.2% (first his‐GFP incubation round saturation: 73.46 pm; second incubation round: 75.79 pm). This indicates a significantly improved signal reproducibility of about a factor of 5. Based on the spectral resolution of our optical characterization setup and the accuracy of the Lorentzian peak‐fitting the experimentally found 3*σ*‐confidence interval of our central lasing mode wavelength determination is ±1 pm (*σ*: standard deviation). From the two subsequently acquired his‐GFP sensing signals we estimated an average detection limit of 159.9 ± 14.6 ng mL^−1^ for the GFP prior signal referencing. After the referencing the detection limit lies at 159.1 ± 5.0 ng mL^−1^. While the absolute detection limit remains virtually unchanged, the confidence interval of the detection limit is diminished from 9.1% to 3.1%. In our estimation we assumed a measuring time of 5 min for the GFP and 7 min for the streptavidin (as performed in Figure 4), respectively. Furthermore, we assumed the sensing signals to scale linearly with protein concentration.^21^ For the streptavidin we deduced a detection limit of approximately 133.9 ± 4.2 ng mL^−1^ from the referenced signal curve shown in Figure 4. While most types of the signal deviations could explicitly be defined by visual inspection of the two lasers signals, some deviations can be interpreted differently. The approximate 3.5 pm redshift found in the Ni‐chelated microlaser (now acting as the reference sensor) during streptavidin incubation (15.5 to 22.5 min) was inferred to be originating from slight refractive index disturbance due to nonspecific adsorption of streptavidin molecules. While this deduction leads to a satisfactory signal referencing, the drift may be alternatively interpreted as a result of a temperature decrease during incubation.

**Figure 4:**
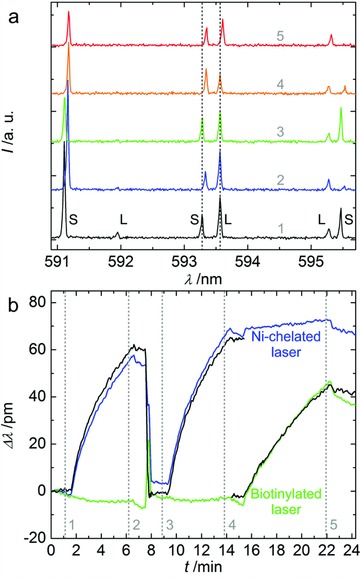
Cross‐referenced simultaneous biomolecular detection and functionalization reconfiguration experiment (small diameter laser: Ni‐chelated, for his‐GFP binding; large diameter laser: biotinylated for streptavidin binding). Injection sequence: 1.5 min: His‐GFP (10 μg mL^−1^ in PBS); 6.5 min: PBS; 7.5 min: Imidazole (125 mmol in PBS); 7.75 min: PBS; 9.25 min: His‐GFP (10 μg mL^−1^ in PBS); 14.25 min: PBS; 15.25 min: Streptavidin (6 μg mL^−1^ in PBS); 22.25 min: PBS. a) FVB spectrum of the microlaser pair (S: lasing mode of small laser, L: lasing mode of large laser) at selected time points 1 to 5 during the experiment (the time points are highlighted in (b)). b) Temporal spectral mode shift of each laser. The interrupted graphs represent the cross‐referenced signals of the individual lasers. To obtain the referenced signal of the biotinylated laser, the Ni‐chelated laser signal is point‐to‐point subtracted from the biotinylated laser's raw signal, and vice versa, under consideration of the temperature and refractive index sensitivities of the individual lasers.

In conclusion, we have demonstrated a sensing platform for straightforward cross‐referenced detection of multiple different molecules, based on the utilization of densely arranged WGM PMMA microgoblet laser pairs in the very same (microfluidic) environment. The close proximity of the lasers enables parallel free space optical pumping and read‐out, avoiding the alignment challenges of tapered fiber coupling. We have shown that the simultaneously acquired spectral signals of the individual lasers can be harnessed for facile but effective mutual referencing. This allows compensation of parasitic signal disturbances originating from temperature and refractive index changes. This is a unique feature not readily available for other single WGM structures, where, e.g., sub‐micrometer sized stray particles have to be introduced with high lateral precision to establish a pure temperature referencing by scattering induced mode splitting.^15^ Additionally, our approach supersedes the very high resolution WGM detection schemes mandatory for resonance discrimination.^1^ Furthermore, we have demonstrated the multiplexing capability by the detection of two different proteins. Besides microring‐based structures with significantly lower Q‐factors, no other on‐chip sensing platform allowing simultaneous interrogation of multiple WGM cavities has yet been demonstrated. We have shown that signal referencing leads to fivefold increased signal reproducibility. We have shown AμCS to provide an unparalleled high lateral precision for selective and contamination‐free functionalization of the individual lasers. We have demonstrated a novel approach for a facile complete chemical reconfiguration of WGM biosensing devices. In contrast to protocols re‐availing the binding receptors for multiple detection of the same type of analyte molecule,^8^ our approach opens up for the first time the possibility to perform multiple completely independent binding experiments, e.g., by subsequently utilizing different acceptor molecules on the same WGM transducer. The reconfiguration is done using microfluidics only and can be performed in closed lab‐on‐a‐chip environments without requiring removal of the sample from the measurement system.^23^ As his‐ and biotin‐conjugates are widespread in protein configurations a large variety of acceptor molecules is readily available. Our results are paving the way for the realization of complex multimolecular analyte experiments, e.g., with blood, which generally suffer from different analyte temperatures, local refractive index fluctutions and nonspecific molecular adsorption. In this regard, the use of phospholipid inks as the surface functionalization might be particularly beneficial, due to the known high protein repulsion. While we have already shown the good passivation capability against the proteins used within this manuscript, and on single microgoblet lasers against bovine serum albumin in our previous publication,^18^ the sensing and signal referencing performance in more complex media like blood plasma still has to be confirmed. Here, particularly lipid‐based analyte components might interact with our surface functionalization, potentially resulting in significant nonspecific signal contributions. Biosensor assemblies (“lipid gratings”) of similar composition as in our present approach were already demonstrated to retain their geometry and specific sensing functionality in fetal calf serum in our lab.^22^ We expect the current biosensor setup to perform equally well.

Our experiments presented here focus on the implementation of two lasers, allowing for the detection of multiple proteins in a sequential manner. To establish simultaneous detection larger laser arrays may be utilized availing at least one entirely passivated microlaser for signal referencing. While surface functionalization could be provided straightforward by multiplexed AμCS,^18^ particularly larger microlaser arrays will require pump spot shaping for effective optical excitation of all microlasers. By the use of a microscope objective with lower magnification and thus a larger field of view, about ten microlasers may be simultaneously read out by our current optical characterization setup.

Within the presented experiments we assumed the signal disturbances to be mainly triggered by the instants of the fluid injections. The type of disturbance for signal referencing was decided on by the user on visual assessment of the temporal signal progression of the two microlasers. The sensing signals were then referenced either for refractive index or for temperature disturbances at a time, not for both simultaneously. Accounting for signal disturbances at any time during measurement could be performed by an in situ (or post) analysis of the time progression of the reference and sensing laser signal. Based on the progression of the reference signal all possible correction factor combinations could then be applied to reconstruct the expected signal progression of the sensing signal. By this procedure different types of disturbances occurring simultaneously (e.g., nonspecific absorption‐based refractive index disturbances superimposed by temperature drifts) would be accounted for.

The presented cross‐referencing and microfluidic reconfiguration capabilities along with the mass‐fabrication compatibility^17^ as well as facile detection schemes open up very promising perspectives for a utilization in portable lab‐on‐a‐chip systems for point of care diagnostics.^20^, ^24^

## Experimental Section

*Microgoblet Laser Fabrication*: The microgoblet laser pairs were fabricated out of pyrromethene 597 (Radiant Dyes) doped PMMA. A 1.2 μm thick PMMA (PMMA 950k A6, MicroChem) layer was spun onto silicon wafers. Subsequently, arrays of microdisc pairs were structured into the PMMA layer by electron beam lithography. The pairs each comprised microdisks with radii *R*_L_ = 25 μm and *R*_S_ = 20 μm, with an edge‐to‐edge distance of 500 nm. After spray development of the PMMA with MIBK:IPA (methyl isobutyl ketone:isopropanol) and isotropic dry etching of the silicon with XeF_2_, the disks were located on silicon pedestals, with free‐standing circumferences. The wafer was then thermally annealed at 125 °C on a hotplate, above the glass transition temperature of PMMA. Due to the along going reduction of the PMMA's surface free energy, the surface roughness of the disks is decreased and the disks form to the characteristic goblet shape. The final edge‐to‐edge distance of the goblets was around 3 μm. Eventually, the wafers were split into chips comprising one laser pair each.

*Aligned Microcontact Stamping [AμCS]*: The AμCS was performed by utilization of a commercial DPN‐system NLP 2000 (NanoInk, USA). The stamp pads were fabricated by pipetting the various phospholipid inks onto standard microscope glass slides. The inks were transferred onto the individual microgoblets at room temperature and 60–70% relative humidity. A detailed description about the AμCS method can be found elsewhere.^18^

*Utilized Phospholipids*: 1,2‐dioleoyl‐*sn*‐glycero‐3‐phosphocholine (DOPC; carrier lipid), 1,2‐dipalmitoyl‐*sn*‐glycero‐3‐phosphoethanolamine‐n‐(cap biotinyl) (Biotinyl Cap PE; biotinylated lipid); 1,2‐dioleoyl‐*sn*‐glycero‐3‐{[*N*(5‐amino‐1‐carboxypentyl)iminodiacetic acid]succinyl} (DOGS‐NTA‐Ni; Ni‐chelated lipid) (all from Avanti Polar Lipids, USA).

*Fabricated Inks*: DOPC admixed with 4 mol% Biotinyl Cap PE (biotin ink), DOPC with 25 mol% DOGS‐NTA‐Ni (Ni‐chelate ink).

*Refractive Index Measurements*: The refractive indexes of the various solutions were measured by an Atago PAL‐RI refractometer (Atago, Japan).
